# Cardiac tamponade complicating thoracocentesis: a case for image-guided procedure

**DOI:** 10.11604/pamj.2018.29.37.14335

**Published:** 2018-01-16

**Authors:** Anthony Adeseye Adeniran, Omolade Oluwafadekemi Adegoke, Akinwumi Oluwole Komolafe

**Affiliations:** 1 Department of Morbid Anatomy and Forensic Medicine, Obafemi Awolowo University Teaching Hospitals Complex, Ile-Ife, Nigeria

**Keywords:** Cardiac tamponade, thoracocentesis, cardiogenic shock

## Abstract

We present a case of cardiac tamponade that was precipitated by thoracocentesis and discovered at autopsy.

## Introduction

Cardiac or pericardial tamponade is a life-threatening condition characterized by the slow or rapid compression of the heart by fluid, pus, blood, clots, or gas, as a result of effusion, trauma, or rupture of the heart, which has accumulated between the visceral and parietal pericardia [1]. Such compression leads to impairment of the pumping action of the heart with subsequent cardiogenic shock [2]. The fluid within the pericardial space may build up suddenly or slowly depending on the aetiology. Cardiac tamponade is a rare and serious complication of thoracocentesis (also known as pleural aspiration). Thoracocentesis is a medical procedure done by inserting a needle and sometimes a plastic catheter through the chest wall to remove fluid from the space between the lungs and the chest wall [3]. We hereby present a case of cardiac tamponade found at autopsy following left sided thoracocentesis. The role of the autopsy and its pivotal role in clinical care are further highlighted via this index case [4-6].

## Patient and observation

**Figure 1:**
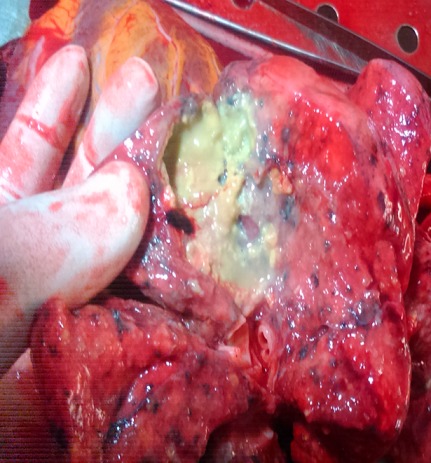
Cut surface of the lower lobe of the left lung showing one of the areas of chronic granulomatous caseous necrosis

**Figure 2:**
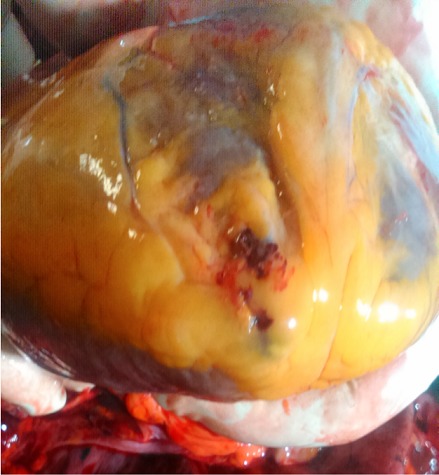
Puncture wounds at the apex of the heart. Note the vital reaction at the site of the injury

**Figure 3:**
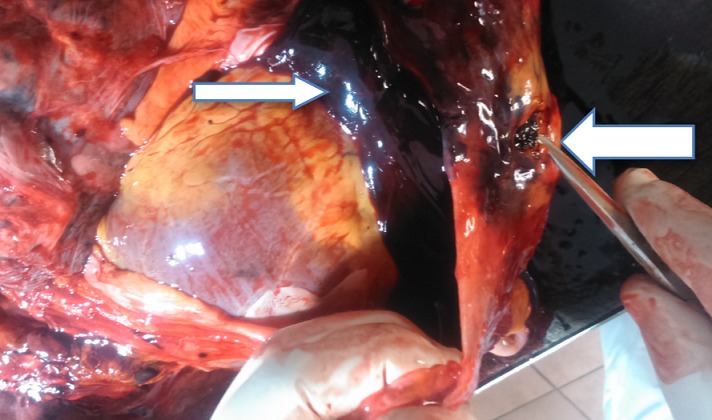
Laceration of the pericardial sac (big arrow); massive blood clot within the pericardial space (small arrow) that led to cardiac tamponade

A 44-year-old woman was admitted into the ward on account of three months history of cough and one-week history of fever and difficulty with breathing. The cough was insidious in onset and productive of yellow sputum that is non-bloody and non-foul smelling. The fever was high grade with associated chills and rigor. There was associated history of progressive weight loss and drenching night sweats. There was no history of contact with person with chronic cough. Physical examination revealed a middle-aged woman who was in obvious respiratory distress. The pulse rate was 92 beats per minute (regular and good volume) with a blood pressure of 100/60mmHg and respiratory rate of 36 cycles per minute. Chest examination revealed dull percussion notes and absent breath sounds on the left lower lobes of the left lung. The apex beat was located in the fifth left intercostal space, mid clavicular line. An assessment of pulmonary tuberculosis was entertained to rule out metastatic lung disease. Chest x-ray that was done showed homogenous opacity with circumscribed nodular lesions of the left lower lung zones. Emergency in-patient thoracocentesis (with no imaging guidance) was performed for microbiological studies and cytology with 10mls of haemorrhagic fluid aspirated. However, some minutes after the procedure, patient complained of dizziness and went into coma. She was pronounced dead after failed resuscitation and autopsy was performed in our facility. Findings at autopsy revealed shrunken and partially fibrotic lower lobe of the left lung (about 60% of normal) with the presence of three areas of caseous necrosis located in both the upper and lower lobes (Figure 1). The enlarged heart (due to hypertensive heart disease) had replaced the space left by the shrunken lung. There was about 400mls of serosanguineous left pleural effusion. There were about four puncture wounds located at the apex of the heart, adjacent to the site of the pericardial laceration (Figure 2). Gross examination of the pericardial sac showed a 1.2 x 0.7cm laceration of the pericardial sac at the apex with about 650mls of clotted blood within the pericardial space (Figure 3). Considering the acuteness or sudden nature of the onset of symptoms and the progressive deceleration to death; the puncture was ascertained to be iatrogenic. The cause of death from autopsy findings was presented as cardiac tamponade due to iatrogenic puncture of the heart in a failed thoracocentesis. Histology of samples taken from the left lung tissue confirmed the presence of chronic caseating granulomatous inflammation due to pulmonary tuberculosis.

## Discussion

Thoracocentesis (pleural aspiration) describes a procedure whereby pleural fluid or air is aspirated via a system inserted temporarily into the pleural space [6]. This may be for diagnostic purposes or therapeutic to relieve symptoms [6, 7]. It is required in many clinical settings for variety of reasons, most especially in emergency situation [6]. Complication is very rare, however those that are commonly encountered include pneumothorax, re-expansion pulmonary oedema and haemorrhage [6, 7]. Serious complication of visceral injury, like cardiac puncture, is rarely encountered [6, 7]. This is quite rare but becomes very significant when it ends in fatality as it occurred as in this case [7]. In this index case, the primary pathology of the left lung, which is secondary pulmonary tuberculosis, a chronic granulomatous inflammatory condition, has caused replacement of lung tissue by fibrocollagenous tissue. This disease of the lung had significantly destroyed the lung tissue, left the lung shrunken; thereby creating a space for the enlarged heart to float inside the pleural fluid. This created a situation whereby the inserted needle caused injury to the pericardial sac and also the heart, precipitating cardiac tamponade, which eventually caused death by cardiogenic shock [2]. It is pertinent to state that the physician who performed the thoracocentesis did the procedure as a blind procedure. A proper appraisal of the imaging procedures (X-ray, CT scan etc) would have cautioned as to the possible challenges of traumatising the enlarged heart which was displaced beyond its presumed confinement. Studies and guidelines have advocated and recommended the use of image-guidance for thoracocentesis [1]. Jones et al found that the complication rate with thoracocentesis performed under ultrasound guidance is lower than that reported for non-image-guided thoracocentesis [8]. In addition to ultrasound-guided thoracocentesis, Havelock et al reiterated the British Thoracic Society pleural disease guideline for adequate and continuous training of doctors (both junior and senior) for effective and efficient mastery of the procedure [6]. Failure to follow explicit guidelines in the management of patients may expose physicians to embarrassing litigations [9, 10].

## Conclusion

Risk of cardiac injury is high with non-image guided thoracocentesis especially in individuals with suspected chronic lung disease. Improvement and strict adherence to laid out guidelines should be implemented. Thoracocentesis should be performed in places where emergency support can be easily mobilized to remedy complications should they occur.

## Competing interests

The authors declare no competing interests.
